# Emergency response planning for sudden cardiac arrest in amateur football clubs in Germany (federal state Saarland)

**DOI:** 10.1136/bmjsem-2024-002274

**Published:** 2025-01-06

**Authors:** Florian Egger, Ana Ukaj, Tim Meyer

**Affiliations:** 1 Institute of Sports and Preventive Medicine, Saarland University, Saarbrucken, Germany; 2 Institute of Sports and Preventive Medicine Campus B8 2, Saarland University, Saarbrucken, Germany

**Keywords:** Football, Resuscitation, Automatic external defibrillator, Death, Soccer

## Abstract

**ABSTRACT:**

**Objective:**

While emergency care for sudden cardiac arrest (SCA) is strictly regulated in professional football, the situation in amateur football is unclear. This study investigated the emergency readiness for SCA in German amateur football clubs.

**Methods:**

A cross-sectional survey of 253 German amateur football clubs (fifth division and lower) was conducted between January and August 2023. Club representatives participated in a 30-point questionnaire on automated external defibrillator (AED) availability, visibility, purchase, usage, frequency of staff trained in cardiopulmonary resuscitation (CPR) and AED usage, regular CPR and AED training, and the existence of an emergency action plan (EAP).

**Results:**

161 of 253 eligible clubs (64% response rate) participated. An AED was available in 48/161 (30%) clubs. 46 of 161 clubs (29%) had no CPR-trained staff. A high availability of CPR- and AED-trained staff (>75% likelihood of being present at the pitch) was more likely during a match (61% and 84%) than training (40% and 51%), respectively. Retrospectively, over 7 years, five clubs reported that CPR-trained staff used an AED, resulting in a survival rate of 80%. 16 clubs (10%) had an EAP in the event of an SCA.

**Conclusion:**

German amateur football clubs show low emergency readiness for SCA despite a promising survival rate when an AED is used by CPR-trained staff on-site. Regular CPR and AED training for club members, increased availability of AEDs, and the development of EAPs might be beneficial in responding adequately to an SCA during football training and matches.

**WHAT IS ALREADY KNOWN ON THIS TOPIC**

- Emergency response planning for sudden cardiac arrest (SCA) seems sufficient in top-division professional football clubs but tends to be inadequate in lower divisions.

**WHAT THIS STUDY ADDS**

- Emergency readiness data from a wide range of amateur football clubs at a national level.
- 80% of clubs have never offered cardiopulmonary resuscitation (CPR) training to their members, 70% of clubs do not provide an automated external defibrillator (AED) and 29% of clubs have no CPR-trained staff.
- A high availability of CPR- and AED-trained staff was more likely during match (61% and 84%) than training (40% and 51%).
- A high SCA survival rate of 80%, even in older football players, can be reached if prompt, high-quality CPR is performed and an AED is used.

**HOW THIS STUDY MIGHT AFFECT RESEARCH, PRACTICE OR POLICY**

- The current results can raise awareness among amateur football clubs and encourage them to provide CPR and AED training for their members to build skills and make their pitch a ‘cardio-protected-area’.

## Introduction

 The leading medical cause of death in athletes is sudden cardiac arrest (SCA).^1 2^ When SCA occurs, shortening the time from collapse to cardiopulmonary resuscitation (CPR) is crucial.^3^ A favourable neurological outcome in out-of-hospital cardiac arrest (OHCA) decreases by 13% for every minute CPR is delayed.^4^ Exercise-related OHCA is associated with a better prognosis than non-exercise-related OHCA as it is more frequently witnessed, resulting in higher rates of bystander CPR, automated external defibrillator (AED) usage and shockable initial rhythm.^5^ In student-athletes, SCA showed a high survival rate (89%) when prompt CPR and early use of an AED were performed.^6^ Similar results were reported for predominantly competitive football players, demonstrating a survival rate of 85%.^7^ These observations strongly support AED availability, regular CPR training and an emergency action plan (EAP) at sports venues.^6 7^ While European top-division football clubs usually ensure emergency response planning during matches and training by relevant regulations,^8^ clubs in lower divisions, especially at amateur and grassroots levels, seem less prepared.^9^,^11^ In Ireland, amateur clubs from various sports (including 28 football clubs) reported an AED availability rate of 81%. However, only half of them could provide AED-trained staff on match day.^10^ In a larger study of amateur football clubs, 32% of 173 Dutch clubs having an AED did not have a person skilled in using the device during training.^11^ Nevertheless, beyond these initial findings, available literature on emergency readiness in amateur football clubs on a national level is limited. Therefore, the present study aimed to investigate the emergency readiness in German amateur football clubs regarding AED availability, the presence of AED-trained staff during matches and training, regular training in CPR and the existence of an EAP.

## Methods

### General design

This cross-sectional study was conducted between January and August 2023 in the German federal state of Saarland. All amateur football clubs (fifth division and lower) registered with the Saarland Football Association (regional chapter of the German Football Association, *Deutscher Fußball-Bund*, DFB) and participating in competitions were considered for inclusion in the study. Professional clubs (first to fourth division) were excluded. Representatives of each amateur club were informed via email. They provided written informed consent to participate in an anonymous 30-item questionnaire (onlinesupplemental materials S1  S2) designed by the authors addressing the following topics: AED availability, visibility, storage, purchase, usage, frequency of CPR and AED trained staff at training or match, regular CPR and AED training, and the existence of an EAP. Reminders were sent every 2 months, four times in total, and when no response was received, the club was finally excluded. The questionnaire contained closed-ended, open-ended and grouped-answer questions. Likert scales were used where appropriate to facilitate subjective responses. Participants were not involved in the planning of our research. We followed the Strengthening the Reporting of Observational Studies in Epidemiology cross-sectional checklist.^12^

### Statistics

When normal distribution was confirmed by a Shapiro-Wilk test, data were reported as mean±SD. Non-normally distributed data were expressed as median and IQRs. Descriptive statistical analyses were performed using SPSS (V.18.0), and the results were presented using GraphPad (V.9.0).

## Results

**Figure 1:**
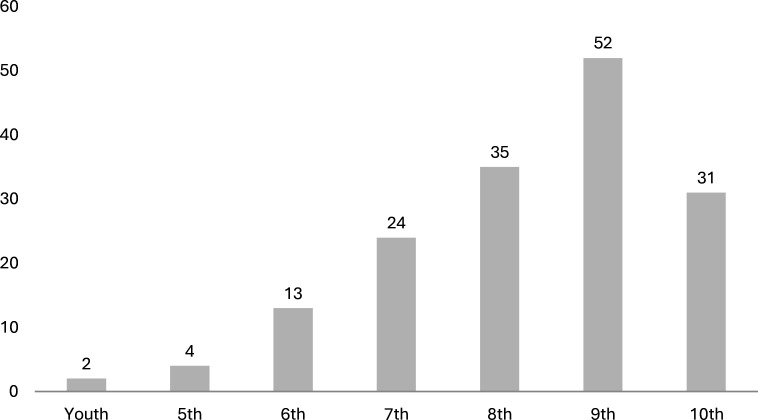
Distribution of the clubs (n=161) across the different amateur leagues.

Of 253 football clubs eligible for this study, 92 did not respond after four inquiries. Finally, 161 of 253 (64% response rate) clubs participated in this study. Respondents were 154 club board members (96%), 4 players (2%) and 3 staff members (2%), with a mean age of 60±10 years and club membership duration of 30±18 years. The distribution of all clubs across the different amateur leagues is presented in figure 1.

### Availability and acquisition of AEDs

In 48 of 161 participating clubs (30%), an AED was available on-site, while 113 clubs (70%) did not have one. For 98 of these 113 clubs (87%) reasons for not providing an AED were reported as follows: high costs (56%, n=55), lack of priority (29%, n=28), unclear (10%, n=10), AED acquisition is already planned (3%, n=3), public AED availability near the venue (2%, n=2). Of the 48 clubs owning an AED, 12 (25%) reported that the time since the acquisition was ≤1 year, 28 (58%) 1 to ≤5 years and 8 (17%) clubs >5 years. The median time since AED acquisition was 2 (IQR: 1.5–4.5) years. Over 7 years, clubs reported five cases of SCA treated with CPR and AED, resulting in a survival rate of 80% (table 1). The AED intervention rate was 1 in 38 club years (cumulative years of AED availability across all clubs divided by the number of cases with AED use).

**Table 1:** Out-of-hospital CPR of five people suffering SCA treated by CPR using an AED by first responders who happened to be at the scene

Year	Gender	Age (years)	Function	Distance to AED (metre)	Moment of CPR	First responder on site	Outcome
2016	Male	55	Player	25	Prompt	Player (physician)	ROSC, survival
2017	Male	35	Player	35	Prompt	Player (physician)	ROSC, survival
2022	Male	75	Spectator	15	Prompt	Coach and player (both trained in CPR)	ROSC, survival
2022	Male	76	Spectator	25	Prompt	Staff member (paramedic) and spectator (nurse)	ROSC died several days later in hospital
2022	Male	68	Player	35	Prompt	Player (trained in CPR)	ROSC, survival

Prompt onset of CPR within a few minutes of SCA and without significant delay. Survival describes an outcome without neurological sequelae.

AEDautomated external defibrillatorCPRcardiopulmonary resuscitationROSC, return of spontaneous circulationSCAsudden cardiac arrest

AEDs were purchased through donations (35%, n=17), a combination of own funds and donations (29%, n=14), own funds (31%, n=15) or from unknown sources (4%, n=2). AED maintenance was performed in 27 (56%), was unknown in 16 (33%) and was not performed in 5 (10%) clubs. All 27 clubs maintaining their AED reported that an inspection had been performed ≤2 years, and 18 (67%) of them ≤1 year.

### Storage and accessibility of AEDs

**Figure 2:**
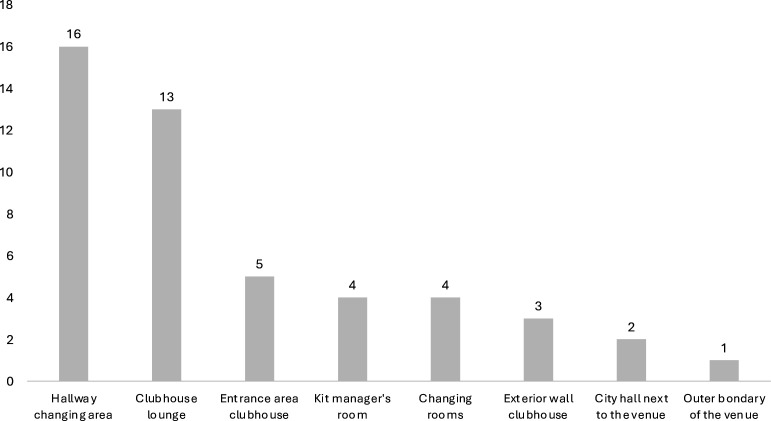
Localisation of the automated external defibrillators in 48 amateur football clubs.

The different localisations of the AEDs reported by the clubs are presented in figure 2. The majority of the AEDs (88% n=42) were stored inside the clubhouse, 8% (n=4) were available outside the venue and 4% (n=2) on the neighbouring premises. AEDs were freely accessible and ready at hand in 45 (94%) clubs and locked in 3 (6%) clubs. The visibility of the AEDs was said to be good (visible to everyone visiting the venue), limited (not visible at first glance) or very limited (only a few members have access and know where the AED is) in 17 (35%), 26 (54%) and 5 (10%) clubs, respectively. The estimated distance from the AED to the pitch (range: 5–100 m) was ≤20 m in 22 clubs (44%), 20 to ≤50 m in 23 clubs (48%) and ≥50 m in three clubs (6%). In 26 of 48 clubs with an AED (54%), training also took place on alternative pitches outside the club grounds, of which 3 (6%) were equipped with an AED.

### CPR and AED-trained club members

**Figure 3:**
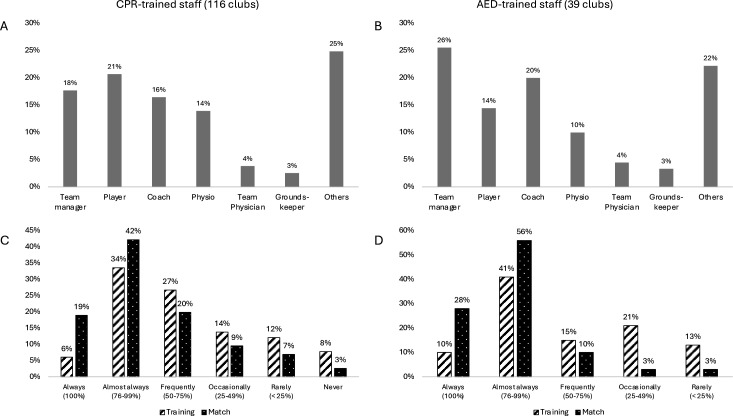
Distribution of different groups of AED-trained staff (**A**) and CPR-trained staff (**B**). Estimated likelihood of being present at the pitch of AED-trained staff (**C**) and CPR-trained staff (**D**) during training and match. AED, automated external defibrillator; CPR, cardiopulmonary resuscitation.

In 29%, 49%, 17% and 6% of all clubs, there were no 1–5, 6–10 and >10 CPR-trained club members, respectively. In 19%, 50%, 19% and 13% of the clubs with AED, there were no 1–5, 6–10 and >10 AED-trained members, respectively. Medical professionals (team physicians and physiotherapists) accounted for only 18% and 14% of CPR- and AED-trained staff, respectively (figure 3). A high availability of CPR- and AED-trained staff (>75% probability of being present at the pitch) was more likely during a match (61% and 84%) than training (40% and 51%), respectively. A lack of CPR- and AED-trained staff (<25% likelihood of being present at the pitch) was more likely during training (20% and 34%) than during the match (10% and 6%), respectively. CPR training occurred annually, greater than or equal to every two years, greater than or equal to every five years or never in 2%, 7%, 11% and 80% of clubs, respectively. A written EAP was present in 10% of clubs, not in 80% of clubs and another 10% did not know.

## Discussion

Although in European professional football, high standards for emergency readiness in the event of SCA are applied,^9 13^ amateur football teams reveal a different picture. This study demonstrates deficits in the emergency response planning of German amateur football clubs. Major links in the out-of-hospital ‘chain of survival’,^14^ such as rapid recognition of SCA, prompt provision of high-quality CPR and defibrillation, are insufficiently in place. This was reflected in 80% of clubs having never offered CPR training to their members, 70% not providing an AED and 29% of clubs having no CPR-trained staff. These figures are concerning, considering that most bystanders are not trained in CPR and do not recognise SCA.^15^ However, if a bystander performs CPR, a recent meta-analysis could clearly show improved survival to hospital discharge.^16^ Moreover, the combination of trained first responders and prompt CPR with an AED has shown survival rates of 85%, 89% and 91% in athletes.^6 7 17^ Notably, under similar circumstances, the survival rate of five cases reported in the present study was 80%.

An EAP, beneficial for a favourable survival rate through structured, practised and rapid response to SCA, was only present in 10% of football clubs investigated in this study. This problem does not seem to be exclusive to amateur football. Similar low proportions of EAPs have been observed in various Australian sports clubs and facilities (12%) and in US amateur basketball teams (6%).^2 18^

Initial observations of emergency readiness in European amateur football from Ireland and the Netherlands showed more favourable findings than those found in the present study.^10 11^ In 50% of 218 Irish amateur clubs (including 28 football clubs) and 84% of 205 Dutch amateur football clubs, AED-trained staff is on-site on match day.^10 11^ In contrast, this only applies to 28% of German amateur football clubs (figure 3). Similar findings were observed for training sessions with higher availability of AED-trained staff in Ireland (30%) and the Netherlands (68%), compared with 10% in Germany. Altogether, previous findings indicate a general shortage of AED-trained staff in amateur football, which has potential for improvement. This problem might be due to ignorance, reluctance or ‘reservedness’ for an AED. Evidence shows that a short educational intervention can significantly improve individuals’ confidence, willingness and knowledge in using AEDs.^19^ Dutch and Irish amateur football clubs showed much higher AED availability rates (97% and 64%) than German football clubs (30%), probably related to different national CPR and AED training strategies and policy requirements. Further reasons for this discrepancy could be that more than half of German clubs reported high costs as a barrier to purchasing an AED, and one-third did not consider purchasing AEDs a priority.

Apart from AED availability, the accessibility of AEDs is decisive for early defibrillation,^20^ which should be performed within the first 3 min of SCA to improve the survival rate.^21^ In this study, 94% of German football clubs who possess AEDs reported freely accessible and ready-to-use AEDs and a distance between the AED and the pitch of no more than 100 m, which shows that at least the formal requirements for early defibrillation are in place in these clubs. However, when training took place on a different pitch, only a small proportion of clubs benefited, as only 6% had an AED available on site. Although the visibility of AEDs was limited in half of the German football clubs, this does not seem to impact delaying defibrillation.^20^ Taken together, the good AED accessibility of German football clubs cannot outweigh the poor AED availability. However, it must be clear that a high AED availability is useless if AEDs are not used. Therefore, the prerequisite for a high SCA survival rate includes the presence of CPR- and AED-trained staff.^6 7 17^ In this context, medical professionals accounted for only low proportions of CPR- and AED-trained staff in German amateur clubs, which is in line with observations in Dutch amateur football.^11^

### Practical implications

A low AED use is not only a concern in the general population,^22^ but also in football and other sports.^7 17^ The most common barriers to CPR and AED initiation are fear of causing harm, feeling incapable of help, difficulty in recognising the signs of SCA and inadequate practice of EAP procedures.^15 23 24^ To address these weaknesses and build competence, the *Fédération Internationale de Football Association* (FIFA) has introduced emergency medical training courses to medical officers, team physicians and physiotherapists in preparation for FIFA competitions. It has also developed field-of-play-specific protocols for recognising and responding to SCA.^25^ Such an approach is also needed for amateur football but would rather be a task for national associations or political institutions. Players, coaches, staff and referees should know how to recognise an SCA to promptly start CPR. In this context, the unnecessary and time-wasting prevention of the ‘swallowed tongue’ of an unconscious player should be clarified as a myth, which is possibly one of the main obstacles to initiating CPR.^26^ In contrast, football players should be made much more aware that if a player collapses and becomes unresponsive on the pitch without prior contact, SCA should be assumed until proven otherwise.^27^ Football players are the most frequent first responders.^7^ Nevertheless, in this study, players only accounted for a maximum of one-fifth of CPR- or AED-trained staff (figure 3). Therefore, regular training of players in CPR and AED usage is crucial.

### Limitations

This study is limited by its cross-sectional design. Nevertheless, a high response rate and a broad representation of amateur clubs from the fifth to tenth league was achieved. Most respondents were key representatives of their clubs with decades of membership, making them as reliable as possible. However, some response bias (eg, socially desirable responses) must be expected. Another limitation might be that only the football clubs of the German federal state of Saarland were investigated. However, the proportions of clubs and player characteristics such as sex and age (male, female, youth) are similarly represented in all regional chapters, according to the German Football Association membership statistics.^28^ Nevertheless, Saarland is one of the German federal states with the lowest per capita gross domestic product, which may have impacted the financial problems of the clubs in affording AEDs with limited transferability to whole Germany. Due to the highly descriptive nature of the questionnaire, a validity test was not carried out.

## Conclusion

In summary, the present study describes the emergency readiness for SCA in German amateur football clubs. It identifies deficiencies in the equipment (availability of AEDs), number of CPR-/AED-trained first responders (more pronounced during training sessions) and the organisational structure (written EAPs). In addition to the increased provision of AEDs, which can be linked to financial barriers, clubs should focus on acquiring life-saving skills for their members to significantly increase the chance of survival without sequelae. Thus, the current results can raise awareness among amateur football clubs and encourage them to provide CPR and AED training for their members to build competence, thereby making their pitch a ‘cardio-protected area’. The ability to recognise SCA and to perform high-quality CPR through basic-life support workshops can be easily learnt through video-based courses and maintained for as long as refresher courses are provided. Very importantly, it is essential that the game’s protagonists, the players, are involved. Low emergency readiness for SCA may not only concern amateur football but may also apply to other amateur sports, with possible differences by region and socioeconomic status. Further studies are needed to uncover these ambiguities.

## supplementary material

10.1136/bmjsem-2024-002274 online supplemental file 1

10.1136/bmjsem-2024-002274 online supplemental file 2

## Data Availability

Data are available upon reasonable request.
