# Supplementary material for: Emergency response planning for sudden cardiac arrest in amateur football clubs in Germany (federal state Saarland)
Source: BMJ Open Sport Exerc Med. 2025 Jan 6;11(1):e002274. doi: 10.1136/bmjsem-2024-002274 (PMC11781088; doi:10.1136/bmjsem-2024-002274)
Supplement: online supplemental file 1 [file bmjsem-11-1-s001.pdf]

„Emergency response planning for sudden cardiac arrest in German amateur football clubs“

1. How many years have you been a member of this club?

Enter your answer

2. How many members does your club have?

Enter your answer

3. In which league does your club’s first men's team play?

Enter your answer

4. What is your function in your club?

- o Board member
- o Coach
- o Player
- o Care giver
- o Normal member (without specific function)
- o Other function

5. Does your club have an automated external defibrillator (AED)?

- o Yes
- o No (->continue with question 13)

6. How was your automated external defibrillator (AED) funded?

- o Own funds
- o Own funds and donations
- o Pure donation
- o I don’t know

7. What are the reasons that your club has not yet purchased an automated external defibrillator (AED)?

Enter your answer

8. Since when does your club have an automatic external defibrillator (AED)?

Enter your answer

9. Where is the automated external defibrillator (AED) located during the match and/or training?

Enter your answer

10. Is your club's automated external defibrillator (AED) regularly maintained?

- Yes
- No
- I don’t know

11. How long has it been since the last maintenance of your club's external automated defibrillator (AED) (specify in weeks, months, years)?

Enter your answer

12. Is your club's automatic external defibrillator (AED) locked?

- Yes
- No (freely accessible)

13. How do you rate the visibility of your automated external defibrillator (AED)?

- Clearly visible (for every visitor of the sports field)
- Limited visibility (not visible at first sight)
- Not visible (only a few club members have access and know where the AED is)

14. Approximately how many meters is the distance from the location of the automatic external defibrillator (AED) to the sideline of the pitch?

Enter your answer

15. How often has your club's automated external defibrillator (AED) been used?

- Not yet
- Once
- Twice
- More than twice

16. In which year(s) was the automated external defibrillator (AED) used in your club?

Enter your answer

17. Did the person survive after using your automated external defibrillator?

- Yes
- No

18. Do the teams of your club (including youth) train at other sports facilities as well?

- Yes
- No

19. If so, how is emergency response planning organized there?

- AED on site
- AED and personnel trained in cardiopulmonary reanimation (CPR) on site
- No AED, but personnel trained in cardiopulmonary reanimation (CPR) on site
- Neither AED nor personnel trained in cardiopulmonary reanimation (CPR) on site

20. Which person in your club is trained in the use of an automated external defibrillator (AED) (multiple answers possible)?

- Nobody
- Groundskeeper
- Physiotherapist
- Team manager
- Player
- Coach
- Team doctor
- Others

21. In this context, what is the total number of persons (if nobody, please enter 0)?
Enter your answer

22. Is one of these persons on site during training sessions?

- Never
- Rarely (less than 25%)
- Occasionally (25-49%)
- Frequently (50-75%)
- Almost always (76-99%)
- Always (100%)
- Not applicable (nobody)

23. Is one of these persons on site during matches?

- Never
- Rarely (less than 25%)
- Occasionally (25-49%)
- Frequently (50-75%)
- Almost always (76-99%)
- Always (100%)
- Not applicable (nobody)

24. Who in your club is trained in cardiopulmonary resuscitation (multiple answers possible)?

- Nobody
- Groundskeeper
- Physiotherapist
- Team manager
- Player
- Coach
- Team doctor
- Others

25. In this context, approximately how many people are there? (if nobody, please enter 0)?

Enter your answer

26. Is one of these persons on site during training sessions?

- Never
- Rarely (less than 25%)
- Occasionally (25-49%)
- Frequently (50-75%)
- Almost always (76-99%)
- Always (100%)
- Not applicable (nobody)

27. Is one of these persons on site during training matches?

- Never
- Rarely (less than 25%)
- Occasionally (25-49%)
- Frequently (50-75%)
- Almost always (76-99%)
- Always (100%)
- Not applicable (nobody)

28. How often is a first aid course held within the club?

- Never
- Once a year
- Every 2 years
- Every 3 years
- Every 4 years
- Every 5 years
- Less than every 5 years

29. When was the last first aid course held within the club (if never, please enter "not applicable")?

Enter your answer

30. Does your club have an emergency action plan, that clearly regulates the procedure in the event of a cardiac arrest?

- o Yes
- o No
- o I don’t know
