# Supplementary material for: Emergency response planning for sudden cardiac arrest in amateur football clubs in Germany (federal state Saarland)
Source: BMJ Open Sport Exerc Med. 2025 Jan 6;11(1):e002274. doi: 10.1136/bmjsem-2024-002274 (PMC11781088; doi:10.1136/bmjsem-2024-002274)
Supplement: online supplemental file 2 [file bmjsem-11-1-s002.pdf]

„Notfallbereitschaft im Falle eines plötzlichen Herztodes in deutschen Fußball-Amateurvereinen“ (German version)

1. Wie viele Jahre sind Sie schon in dem Verein Mitglied?

Ihre Antwort eingeben

2. Wie viele Mitglieder hat ihr Verein?

Ihre Antwort eingeben

3. In welcher Liga spielt die 1. Herrenmannschaft Ihres Vereins?

Ihre Antwort eingeben

4. Was ist Ihre Funktion im Verein?

- o Vorstandsmitglied
- o Trainer
- o Spieler
- o Betreuer
- o Normales Mitglied (ohne spezifische Funktion)
- o Andere Funktion

5. Verfügt Ihr Verein über einen automatisierten externen Defibrillator (AED)?

- o Ja
- o Nein (-> fahren Sie mit Frage 13 fort)

6. Wie wurde Ihr automatisierter externer Defibrillator (AED) finanziert?

- o Eigene Mittel
- o Eigene Mittel und Spenden
- o Reine Spende
- o Keine Ahnung

7. Was sind die Gründe dafür, dass Ihr Verein bisher noch keinen automatisierten externen Defibrillator (AED) angeschafft hat?

Ihre Antwort eingeben

8. Seit wann verfügt Ihr Verein über einen automatisierten externen Defibrillator (AED)?

Ihre Antwort eingeben

9. Wo befindet sich der automatisierte externe Defibrillator (AED) während des Spiels und/oder Trainings?

Ihre Antwort eingeben

10. Wird der automatisierte externe Defibrillator (AED) Ihres Vereins regelmäßig gewartet?

- Ja
- Nein
- Ich weiß es nicht

11. Wie lange ist die letzte Wartung des externen automatisierten Defibrillators (AED) Ihres Vereins her (bitte in Wochen, Monaten oder Jahren angeben)?

Ihre Antwort eingeben

12. Ist der automatisierte externe Defibrillator (AED) Ihres Vereins abgesperrt?

- Ja
- Nein (frei zugänglich)

13. Wie schätzen Sie die Sichtbarkeit Ihres automatisierten externen Defibrillators (AED) ein?

- Sehr gut sichtbar (für jeden Besucher des Sportplatzes)
- Eingeschränkt sichtbar (nicht auf den ersten Blick sichtbar)
- Nicht sichtbar (nur wenige Vereinsmitglieder haben Zugang und wissen, wo sich der AED befindet)

14. Wie viele Meter sind es ungefähr vom Ort des automatisierten externen Defibrillators (AED) zum Spielfeldrand?

Ihre Antwort eingeben

15. Wie oft kam der automatisierte externe Defibrillator (AED) Ihres Vereins schon zum Einsatz?

- Noch nie
- Einmal
- Zweimal
- Mehr als zweimal

16. In welchem Jahr (welchen Jahren) kam der automatisierte externe Defibrillator (AED) in Ihrem Verein zum Einsatz?

Ihre Antwort eingeben

17. Überlebte die Person nach dem Einsatz Ihres automatisierten externen Defibrillators (AED)?

- o Ja
- o Nein

18. Trainieren die Mannschaften Ihres Vereins (inklusive Jugend) auch an anderen Sportplätzen?

- o Ja
- o Nein

19. Falls ja, wie ist die Notfallversorgung dort organisiert?

- o AED vor Ort
- o AED und in Reanimation geschultes Personal vor Ort
- o Kein AED, aber in Reanimation geschultes Personal vor Ort
- o Weder AED noch in Reanimation geschultes Personal vor Ort

20. Welche Person in Ihrem Club ist im Umgang mit einem automatisierten externen Defibrillator (AED) geschult (mehrere Antworten möglich)?

- o Keine Person
- o Platzwart
- o Physiotherapeut
- o Betreuer
- o Spieler
- o Trainer
- o Mannschaftsarzt
- o Andere

21. Wieviele Personen sind das insgesamt (falls keine Person bitte 0 eintragen)?
Ihre Antwort eingeben

22. Ist eine dieser Personen während dem Training vor Ort?

- o Nie
- o Selten (weniger als 25%)
- o Gelegentlich (25-49%)
- o Häufig (50-75%)
- o Fast ständig (76-99%)
- o Immer (100%)
- o Nicht zutreffend (keine Person)

23. Ist eine dieser Personen während Spielen vor Ort?

- Nie
- Selten (weniger als 25%)
- Gelegentlich (25-49%)
- Häufig (50-75%)
- Fast ständig (76-99%)
- Immer (100%)
- Nicht zutreffend (keine Person)

24. Wer in Ihrem Verein ist in Reanimation geschult (mehrere Antworten möglich)?

- Keine Person
- Platzwart
- Physiotherapeut
- Betreuer
- Spieler
- Trainer
- Mannschaftsarzt
- Andere

25. Wie viele Personen sind das ungefähr (falls keine Person bitte 0 eintragen)?

Ihre Antwort eingeben.

26. Ist eine dieser Personen während dem Training vor Ort?

- Nie
- Selten (weniger als 25%)
- Gelegentlich (25-49%)
- Häufig (50-75%)
- Fast ständig (76-99%)
- Immer (100%)
- Nicht zutreffend (keine Person)

27. Ist eine dieser Personen während Spielen vor Ort?

- Nie
- Selten (weniger als 25%)
- Gelegentlich (25-49%)
- Häufig (50-75%)
- Fast ständig (76-99%)
- Immer (100%)
- Nicht zutreffend (keine Person)

28. Wie oft wird ein Erste-Hilfe-Kurs vom Verein organisiert?

- Nie
- Einmal pro Jahr
- Alle 2 Jahre
- Alle 3 Jahre
- Alle 4 Jahre
- Alle 5 Jahre
- Seltener als alle 5 Jahre
-

29. Wann fand der letzte Erste-Hilfe-Kurs vereinsintern statt (falls nicht, bitte „Nicht zutreffend“ ankreuzen)?

Ihre Antwort eingeben.

30. Gibt es einen Notfallplan im Verein, welcher die klaren Abläufe bei einem Herzstillstand regelt?

- Ja
- Nein
- Keine Ahnung
